# MR-based attenuation correction in brain PET based on UTE sequences

**DOI:** 10.1186/2197-7364-1-S1-A35

**Published:** 2014-07-29

**Authors:** Jorge Cabello, Stephan G Nekolla, Sibylle I Ziegler

**Affiliations:** Department of Nuclear Medicine, Klinikum rechts der Isar, Technische Universität München, Kragujevac, Germany

Attenuation correction (AC) in brain PET/MR has recently emerged as one of the challenging tasks in the PET/MR field. It has been shown that to ignore the attenuation produced by bone can lead to errors ranging from 5-30% in regions close to bone structures. Since the information provided by the MR signal is not directly related to tissue attenuation, alternative methods have to be developed. Signal from bone tissue is difficult to measure given its short transverse relaxation time (T2). Ultrashort-echo time (UTE) pulse sequences were developed to measure signal from tissues with short T2. A combination of two consecutive UTE echoes has been used in several works to measure signal from bone tissue. The first echo is able to measure signal from bone tissue in addition to soft tissue, while the second echo contains most of the soft tissue contained in the first echo but not bone. In this work we extract the attenuation information from the difference between the logarithm of two images obtained after applying two consecutive UTE pulse sequences using the mMR scanner (Siemens Healthcare). Subsequently, image processing techniques are applied to reduce the noise and extract air cavities within the head. The resulting image is converted to linear attenuation coefficients, generating what is known as µ-map, to be used during reconstruction. For comparison purposes PET/CT scans of the same patients were acquired prior to the PET/MR scan. Additional µ-maps obtained for comparison were extracted from a Dixon sequence (used in clinical routine) and an additional µ-map calculated by the scanner based on UTE pulse sequences. Preliminary quantitative results measured in the cerebellum, using the value obtained with CT-based AC as reference, show differences of 34% without AC, 13% using the Dixon-based and UTE-based provided by the scanner, and 0.8% with the AC strategy presented here.

